# The burden of hyponatremia and 30-day outcomes among adults admitted with stroke at a large tertiary teaching hospital in Northwestern Tanzania

**DOI:** 10.3389/fstro.2025.1546358

**Published:** 2025-03-21

**Authors:** Johari Katanga, Igembe Nkandala, Joshua Ngimbwa, Lilian Andrew Mwamba, Innocent Kitandu Paul, Sospeter Berling, Gladness Xavier, Matilda K. Basinda, Sophia Kagoye, Karim Mahawish, Sarah Shali Matuja

**Affiliations:** ^1^Department of Internal Medicine, Catholic University of Health and Allied Sciences-Weill Bugando School of Medicine, Mwanza, Tanzania; ^2^Department of Internal Medicine, Aga Khan University, Dar es Salaam, Tanzania; ^3^Department of Internal Medicine-Neurology, Jinzhou Medical University, Jinzhou, China; ^4^National Institute for Medical Research, Mwanza Research Centre, Dar es Salaam, Tanzania; ^5^Department of Stroke Medicine, Counties Manukau Health, Auckland, New Zealand

**Keywords:** stroke, hyponatremia, morbidity, mortality, hypertension, Tanzania

## Abstract

**Background:**

The most frequent electrolyte derangement in adults with stroke is hyponatremia, which is associated with increased morbidity, mortality, and prolonged hospital stay. The study aimed to investigate the hyponatremia incidence and 30-day outcomes among adults admitted with stroke at a large tertiary teaching hospital in Northwestern Tanzania.

**Methods:**

This cohort study recruited adults presenting with first-ever stroke (as defined by the World Health Organization) between November 2023 to May 2024. Data were collected on demographics, the degree of neurological impairment at admission using the National Institutes of Health Stroke Scale (NIHSS), and laboratory workup, including sodium levels, on admission; the modified Rankin Scale was used to assess stroke outcomes. We used modified Poisson and logistic regressions to examine factors associated with hyponatremia and 30-day outcomes, respectively.

**Results:**

In total, 167 adults were enrolled, of which 56.9% (*n* = 95) were female, with a median age of 60 years (interquartile range [IQR] 40–74), and 71.2% (*n* = 119) had hypertension and heart failure. The hyponatremia incidence was 29.3% (*n* = 49), and among these participants, 53% (*n* = 26) had mild hyponatremia. Factors associated with hyponatremia were the use of mannitol on admission (adjusted prevalence ratio [aPR] 3.14, 95% CI [1.81, 5.44], *p* < 0.001) and increasing NIHSS scores (aPR 1.03, 95% CI [1.00, 1.06], *p* < 0.05). There were no differences in 30-day mortality between those with and without hyponatremia (respectively, 38.3% vs. 36.7%, *p* = 0.79). The presence of leukocytosis was independently associated with 30-day mortality (adjusted odds ratio [aOR] = 2.7, 95% CI [1.39, 5.36], *p* = 0.004), and the median length of hospital stay was significantly higher in those with hyponatremia compared to those without: 7 days (IQR 4–9) vs. 5 days (IQR 3–9), *p* = 0.032.

**Conclusion:**

Hyponatremia, which is associated with increased stroke severity, probable infections, and prolonged hospital stays, is prevalent among adults with stroke in Northwestern Tanzania. The high prevalence of hypertension and heart failure underscores the need for targeted preventive strategies. Early detection and appropriately managing hyponatremia are essential to improve stroke outcomes in this region.

## Introduction

Stroke is the third-leading cause of death and the fourth-leading cause of disability-adjusted life years (DALYs) globally, with the majority of this burden (over 90% of deaths and DALYs) observed in low- and middle-income countries, particularly sub-Saharan Africa (SSA) (Feigin et al., 2022, 2021). The incidence and mortality rates of stroke in SSA are rising, largely due to the high prevalence of both modifiable and non-modifiable risk factors, such as age, gender, hypertension, and diabetes (Zhang et al., 2010; Abissegue et al., 2024). Studies in SSA indicate a growing burden of stroke, with crude incidence rates increasing from an average of 53 cases per 100,000 between 1973 and 1991 to 88 cases per 100,000 between 2003 and 2011 (Chukwudelunzu, 2024). Notably, in this region, stroke disproportionately affects younger individuals and is often associated with poor outcomes due to infections and other medical complications during the acute phase (Matuja et al., 2020, 2023). Among these complications, electrolyte imbalances are particularly common and can significantly worsen clinical outcomes (Hossain et al., 2023). Common causes of hyponatremia include the syndrome of inappropriate antidiuretic hormone secretion and the use of certain anti-hypertensive medications, such as diuretics and dietary salt restrictions for hypertension management, with cerebral salt wasting syndrome being the least common (Atila et al., 2021; Karunanandham et al., 2018; Ehtesham et al., 2019). Furthermore, serum creatinine levels play a crucial role in differentiating between hypervolemic hyponatremia and euvolemic hyponatremia. Research indicates that even small percentage changes in serum creatinine, specifically changes of ≥10% or ≤ -3%, can accurately classify hyponatremic patients (Gabriel Ruiz-Sánchez et al., 2022).

Tanzania, a country in SSA, has reported a high stroke burden in both community- and hospital-based studies. A large community-based study conducted between 2004 and 2006 identified an age-adjusted stroke incidence of 315.9 per 100,000 person-years in urban areas, with a 28-day mortality of 24% (Walker et al., 2010, 2013). Moreover, hospital-based studies have documented 30-day stroke mortality rates ranging from 40.8% to 61.3%, with the highest mortality occurring within the first week of hospital admission (Matuja et al., 2020; Okeng'o et al., 2017). Despite these alarming statistics, data addressing the burden and impact of hyponatremia on stroke outcomes in SSA, including Tanzania, are limited. This study aimed to investigate the hyponatremia incidence and 30-day outcomes among adults admitted with stroke at a large tertiary teaching hospital in Northwestern Tanzania.

## Materials and methods

### Study design and population

This cohort study was conducted at a large tertiary teaching hospital, Bugando Medical Center (BMC), in Northwestern Tanzania between November 2023 and May 2024. BMC offers specialized care to in- and outpatients from all over the country, a catchment of 15 million people, particularly those residing along the shores of Lake Victoria. The hospital has a 1,080-bed capacity, including 150 beds in the medical ward. BMC has a newly established stroke unit; however, treatment is currently limited to secondary preventive measures due to the lack of acute revascularization therapies. Adults (≥18 years) presenting with a World Health Organization (WHO) definition of first-ever stroke were consecutively recruited after obtaining written informed consent from the patient or next of kin (for those unable to consent due to stroke-related disabilities).

### Data collection

Data were collected and managed using an electronic data-capturing database developed and hosted by Vervig^®^ (https://vervig.com/en-US). Information collected included demographics, such as sex, age, residency, marital status, level of education, and at least three available mobile numbers from the patient and next of kin for follow-up purposes. We also recorded any relevant previous or current prescriptions (e.g., for hypertension, diabetes mellitus, and heart diseases), the use of other medications (e.g., non-steroidal anti-inflammatory and anti-seizure drugs), and dietary restrictions (e.g., salt intake) and inquired about the participant's history of smoking and alcohol consumption. Drugs given on arrival were also documented, including the use of mannitol, 3% saline, and half-strength normal saline.

#### Physical examination

This included an assessment of blood pressure, pulse rate, rhythm, and temperature. Blood pressure readings were taken using a standard digital sphygmomanometer, using Micro life BP A50 (Micro life AG, Switzerland), and three readings were taken 5 min apart. Hypertension was defined as systolic blood pressure (SBP) ≥140 mmHg and/or diastolic blood pressure (DBP) ≥90 mmHg or previous/current use of anti-hypertensive medications (Chobanian et al., 2003). The waist–hip ratio (WHR) was calculated as waist circumference divided by hip circumference. Waist and hip circumferences were measured according to WHO recommendations, and WHR (≥0.90 for men and ≥0.85 for women) were classified as obese following WHO guidelines (Streng et al., 2018; Nishida et al., 2010).

#### Laboratory workup

We aseptically collected 15 ml of venous blood for chemistry analysis (Sysmex 1000 machine) within 24 h of admission. The baseline sodium levels were measured before the administration of mannitol, 3% saline, and half-strength normal saline, using the indirect calibration measurement. We also measured urea, creatinine, and lipid profiles, and the results were recorded in specific case report forms (CRFs). Hyponatremia was defined as a sodium level < 135 mEq/l, and the severity of hyponatremia was further sub-classified as mild (130–134 mEq/l), moderate (125–129 mEq/l), and severe (< 125 mEq/l). A urine analysis was done to obtain urine-specific gravity, and the results were recorded in the CRF. Hypervolemia assessment included symptoms suggestive of congestion (dyspnea and reduced urine output) and physically assessing vital signs (blood pressure and pulse rate), signs of raised jugular venous pressure, ascites, pleural effusion, and lower limb edema in keeping a diagnosis of heart failure using the Framingham criteria and additional stigmata of chronic liver disease (Segal, 2017; Mckee et al., 1971). Clinically, euvolemia was defined as the absence of signs of hypovolemia, such as tachycardia, decreased skin turgor, and a dry mucous membrane, and the absence of signs of congestion (Verbalis et al., 2013). Hypovolemic hyponatremia was defined as patients with sodium levels < 135 mEq/l, with volume depletion assessed by clinical evaluation; hypervolemic hyponatremia in patients with readily recognizable conditions (e.g., heart failure and hepatic or renal disease) and volume overload on clinical assessment and euvolemic hyponatremia were those with hyponatremia and equivocal volume status (Lewis, 2023). Serum osmolality was measured as an expression of the total number of particles in a given weight of solvent; the equation used to calculate serum osmolality was 2 × (Na + K) + urea + glucose (Worthley et al., 1987).

#### Brain imaging

A non-contrasted brain computed tomography (CT) scan, acquired on a 128-slice CT scanner (Siemens Somatom Perspective, Siemens Healthcare GmbH, Germany) was performed on all adults with stroke during admission, and the images were interpreted by a radiologist. Ischemic stroke was classified according to the Trial of ORG 10172 in Acute Stroke Treatment (TOAST) criteria, and for those with hemorrhagic stroke, the hematoma location was documented.

Stroke severity was assessed using the National Institutes of Health Stroke Scale (NIHSS) on admission, and the outcomes were assessed using the modified Rankin Scale (mRS) by following up with adult stroke survivors or their next of kin via telephone interviews 30 days after the date of admission (Lewis, 2023; Banks and Marotta, 2007).

### Statistical analysis

The data analysis was performed using STATA version 16. Continuous variables are summarized and presented as a means and standard deviation (*SD*) or median and interquartile range (IQR) depending on the distribution. Categorical variables were summarized as frequencies and proportions. Proportions were compared using Pearson's chi-square or Fisher's exact test as appropriate. The modified Poisson regression with a robust variance estimator was used to identify factors associated with hyponatremia and stroke. Because the outcome variable (hyponatremia) had a proportion of >10%, the analysis using logistic regression overestimated the odds ratio (Supplementary Table 1) (Zou, 2004; Barros and Hirakata, 2003). The factors for the multivariable analyses were selected based on prior knowledge of the possible associations between stroke and hyponatremia, including age and previous medications. Covariates with a *p* < 0.2 in the bivariable analysis were included in the multivariate analysis. The selection of variables that fit in the final multiple regression model was done through a stepwise backward elimination method, which we started by running the multiple regression model with all the predictor variables and then removing those with highest *p*-values one by one from the model until only the predictor variables that best predict the outcome remained in the model. Finally, the best-fit model was selected based on Akaike's information criterion (AIC) for the competing models. The model with the smallest AIC values, compared to the other models, was chosen. Unadjusted and adjusted prevalence ratios, 95% confidence intervals (CIs), and the corresponding *p*-values were obtained from the models. In addition, we performed a logistic regression analysis to examine independent factors associated with 30-day outcomes. In all analyses, a *p* < 0.05 was considered statistically significant.

## Results

**Figure 1:**
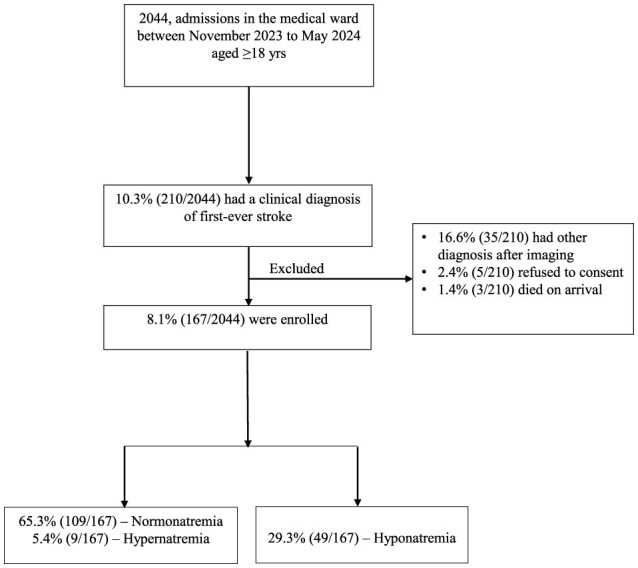
Recruitment flowchart.

There were 2,044 medical admissions between November 2023 to May 2024. Among these, 10.3% (*n* = 210) of adults met the WHO clinical diagnosis for first-ever stroke. We excluded 43 adults for the following reasons: 16.6% (*n* = 35) had stroke mimics, 2.4% (*n* = 5) refused to consent and 1.4% (*n* = 3) died on arrival before consent was obtained. The remaining 79.5% (*n* =1 67) were included in the final analysis. The incidence of adults with hyponatremia was 29.3% (*n* = 49), as seen in Figure 1. Among those with hyponatremia, 53% (*n* = 26/49), 24.5% (*n* = 12/49), and 22.5% (*n* = 11/49) had mild, moderate, and severe severity, respectively.

### The demographic, clinical, and laboratory characteristics of the study cohort

Table 1 summarizes the demographic, clinical, and laboratory characteristics of adults with stroke. The median age was 60 years (IQR, 50–74), and 56.9% (*n* = 95) were females. The proportion of ischemic and hemorrhagic strokes was 59.3% (*n* = 99) and 40.7% (*n* = 69), respectively. Among adults with ischemic stroke, large artery disease was the predominant subtype, 55.6% (*n* = 55). Among those with hemorrhagic stroke, 61.3% (*n* = 46) had intracerebral hemorrhage. Premorbid hypertension and heart failure was observed in 71.2% (*n* = 119) and 65.9% (*n* = 110) were on regular anti-hypertensive medications. The median arrival NIHSS score was 26 (IQR, 13–35), and mannitol was prescribed in 34.1% (*n* = 57) of adults on admission.

**Table 1:** Demographic, clinical, and laboratory characteristics of the study cohort, *N* = 167.

**Variable**	**Frequency, *N* = 167**	**Percentage**
**Age (years)**		
Median (IQR)	60 (50–74)	
**Sex**		
Female	95	56.9
**Marital status**		
Ever married/cohabiting	151	90.4
**Education level**		
No formal education	22	13.2
Primary education	85	50.9
Secondary and above	60	35.9
**Referral status**		
Referral from other facility	109	65.3
**Insurance**		
Not insured	110	65.7
**Occupation**		
Employed	77	46.1
**Alcohol**		
Never	139	83.3
**Smoking**		
Never	151	90.4
**Comorbidities**		
Hypertension and heart failure	119	71.2
Diabetes mellitus	7	4.2
Chronic kidney disease	5	3
Coronary heart disease	24	14.4
Valvular heart disease	8	4.8
Sickle cell disease	4	2.4
**Medication used**		
Antihypertensive	110	65.9
NSAIDS	3	1.8
Antidepressant	1	0.6
Oral hypoglycaemic	48	28.7
Others	5	3
**Salt restriction**		
Restricted	71	42.5
**Medication given on arrival**		
Mannitol	57	34.1
Others^*^	110	65.9
**Pyrexia on admission**		
Yes	155	92.8
**Systolic blood pressure (mmHg)**		
Median (IQR)	148 (124–172)	
**Diastolic blood pressure (mmHg)**		
Median (IQR)	89 (78–101)	
**Waist/hip ratio**		
Increased	165	98.8
Mean (*SD*)	1.37 ± 0.13	
**GCS on admission**		
< 8	54	32.3
9–12	24	14.4
>12	89	53.3
Median (IQR)	13 (8–15)	
**NIHSS score on admission**		
Mild stroke (0–4)	4	2.4
Moderate stroke (5–20)	64	38.3
Severe stroke (≥21)	99	59.3
Median (IQR)	26 (13–35)	
**Type of stroke**		
Hemorrhagic stroke	75	44.9
Ischemic stroke	92	55.1
**Types of hemorrhagic stroke (*****n*** = **68)**		
ICH	46	61.3
IVH	20	26.7
SAH	9	12
**Types of ischemic stroke (*****n*** = **99)**		
Large vessel	55	55.6
Cardio embolic	10	10.1
Small vessel	21	21.2
Other determined etiology	6	6
Undetermined etiology	7	7.1
**Symptom onset to imaging (days)**		
Median (IQR)	2 (1–4)	
**Symptom onset to ED arrival (days)**		
Median (IQR)	2 (1–4)	
**Sodium levels on admission**		
Hypernatremia	9	5.39
Normal	109	65.27
Hyponatremia	49	29.3
**Types of hyponatremias (*****n*** = **49)**		
Euvolemic	19	38.8
Hypervolemic	30	61.2
**Osmolality (*****n*** = **49)**		
Low	31	63.3
Normal	13	26.5
High	5	10.2
**Creatinine (mmol/L)**		
Raised	63	37.7
**Random glucose levels (mmol/L)**		
Median (IQR)	6.7(5.8–7.5)	
**Urea (mmol/L)**		
Raised	57	34.1
**Low-density lipoprotein levels (mmol/L)**		
Raised	16	9.6
**Total cholesterol levels (mmol/L)**		
Raised	61	36.5

IQR, interquartile range; GCS, Glasgow coma scale; NIHSS, National Institutes of Health Stroke Scale; ICH, intracranial hemorrhage; IVH, intraventricular hemorrhage; SAH, subarachnoid hemorrhage; NSAID, non-steroid anti-inflammatory drug; ED, emergency department.

^*^Includes 3% saline, half-strength normal saline, and sodium chloride.

### Factors associated with hyponatremia among the study cohort

Table 2 summarizes the factors associated with hyponatremia among adults with stroke. In the adjusted analysis using the modified Poisson's regression, significant predictors of hyponatremia included the use of mannitol on admission (adjusted prevalence ratio [aPR] = 3.14, 95% CI [1.81, 5.44], *p* < 0.001) and higher NIHSS scores (aPR = 1.03, 95% CI [1.00, 1.06], *p* = 0.046). However, the logistic regression analysis using the same variables did not identify any factors significantly associated with hyponatremia (see Supplementary Table 1).

**Table 2:** Factors associated with hyponatremia.

**Variable**	**CPR [95%CI]**	***p*-value**	**APR [95%CI]**	***p*-value**
Age(years)	1.00 [0.99, 1.01]	0.874	1.00 [0.99, 1.02]	0.194
**Sex**				
Male	Ref			
Female	1.20 [0.73, 1.94]	0.471		
**Comorbidities**				
Hypertension	0.989 [0.56, 1.75]	0.971		
Diabetes	0.92 [0.31, 2.72]	0.877		
Others	Ref			
**Previous medication**				
Hyponatremia-associated^*^	1.13 [0.67, 1.91]	0.649	1.05(0.64-1.72)	0.845
Others^**^	Ref		Ref	
**Medication on arrival**				
Mannitol	3.32 [2.04, 5.40]	< 0.001	3.14 [1.81, 5.44]	< 0.001
Others	Ref		Ref	
**Referral status**				
Self-referral	1.19 [0.73, 1.92]	0.477		
Others	Ref			
**Health insurance**				
Insured	0.9 [0.56, 1.55]	0.791		
Not insured	Ref			
**Alcohol use**				
Yes	1.11 [0.61, 2.04]	0.718		
No	Ref			
**Smoking status**				
Yes	1.07 [0.49, 2.32]	0.859		
No	Ref			
**Salt restriction**				
Yes	1.19 [0.74, 1.91]	0.456		
No	Ref			
**Pyrexia**				
Yes	1.19 [0.43, 3.27]	0.740		
No	Ref			
**Type of stroke**				
Hemorrhagic	1.79 [1.11, 2.86]	0.016	1.02 [0.63, 1.66]	0.927
Ischemic	Ref		Ref	
NIHSS score on arrival	1.01[0.99, 1.04]	0.154	1.03 [1.00, 1.06]	0.046

CPR, crude prevalence ratio; CI, confidence interval; APR, adjusted prevalence ratio; NIHSS, National Institutes of Health Stroke Scale.

^*^Medications included antihypertensives, antidepressants, and non-steroidal anti-inflammatory drugs.

^**^Medications included antidiabetic, xanthine oxidase inhibitors, neuro-support, and others.

### A comparison of 30-day outcomes in adults with stroke with and without hyponatremia

Table 3 presents the 30-day outcomes of adults with stroke based on sodium levels. The overall 30-day mortality rate was 38.3% (*n* = 64), with no statistically significant difference between individuals with and without hyponatremia. Likewise, based on volume status, mortality rates did not significantly differ among adults with hyponatremia, with 42.1% (8/19) in those with euvolemia and 33.3% (10/30) in those with hypervolemia (*p* = 0.374). However, adults with hyponatremia had a longer hospital stay, with a median duration of 7 days (IQR 4–9) compared to 5 days (IQR 3–9) in those without hyponatremia (*p* = 0.032).

**Table 3:** A comparison of 30-day outcomes in adults with stroke with and without hyponatremia.

**Variable**	**Total *N* (%)**	**Hyponatremia *N* (%)**	**No Hyponatremia *N* (%)**	***p*-value**
Median (IQR) length of hospital stay (in days)	5 (5–7)	7 (4–9)	5 (3–9)	0.032
Died at 30 days	64 (38.3)	18 (36.7)	46 (38.9)	0.786
Independency at 30-days (mRS score 0–2)	10 (5.9)	2 (4.08)	8 (6.8)	0.503
Dependency at 30 days (mRS score ≥3)	157 (94.1)	47 (95.92)	110 (93.2)	

IQR, interquartile range; mRS, modified Rankin Scale.

### Factors associated with 30-day mortality among the study cohort

Table 4 describes the factors associated with 30-day mortality. In the multivariate analysis, the presence of leukocytosis was independently associated with mortality (adjusted odds ratio [aOR] = 2.7, 95% CI [1.39, 5.36], *p* = 0.004).

**Table 4:** Factors associated with 30-day mortality.

**Variable**	**Unadjusted OR [95% CI]**	***p*-value**	**Adjusted OR [95% CI]**	***p*-value**
**Sex**				
Female	1.7 [0.99, 3.12]	0.103	1.76 [0.88, 3.48]	0.105
Male	Ref			
Age	1.0 [0.98, 1.11]	0.894		
**Type of stroke**				
Hemorrhagic	1.3 [0.71, 2.55]	0.363		
Ischemic	Ref			
**Hyponatremia**				
Yes	1.1 [0.51, 2.06]	0.935		
No	Ref			
**Health insurance**				
No	0.62 [0.31, 1.22]	0.169	0.67 [0.33, 1.37]	0.275
Yes	Ref			
NIHSS score on arrival	0 (0)	0.999		
Referred	0.55 [0.28, 1.1]	0.088	0.56 [0.27, 1.15]	0.115
Self-referral	Ref			
**Pyrexia**				
Yes	6.7 [0.73, 61]	0.093	2.7 [0.26, 27]	0.408
No	Ref		Ref	
**Hyperglycemia**				
Yes	1.6 [0.50, 5.32]	0.412		
No	Ref			
**Leucocytosis**				
Yes	3.0 [1.6, 5.8]	0.001	2.7 [1.39, 5.36]	0.004
No	Ref		Ref	
**Previous stroke**				
Yes	3.9 [0.47, 33.84]	0.206		
No	Ref			
**Arrival BP** ≥**140/90**				
Yes	0.90 [0.47, 1.74]	0.768		
No	Ref			
**Tachycardia**				
Yes	1.02 [0.51, 2.05]	0.955		
No	Ref			

OR, odds ratio; CI, confidence interval; NIHSS, National Institutes of Health Stroke Scale; BP, blood pressure.

## Discussion

This study aimed to investigate the burden and outcomes of hyponatremia among adults with stroke admitted at a large tertiary teaching hospital in Northwestern Tanzania. We found a high hyponatremia incidence in the young adult population who had preexisting hypertension, heart failure, and poor outcomes at 30 days.

The incidence of adults with hyponatremia in this study was 29.3%, which is significantly higher than the 15.6% and 11.6% reported in studies from Germany and Taiwan, respectively (Huang et al., 2012; Shima et al., 2020). This variation may be attributed to differences in the studies' populations, as the German and Taiwanese studies excluded patients who had received mannitol and hypertonic saline. In addition, the German study focused exclusively on hemorrhagic strokes, while the Taiwanese study included only ischemic strokes. Notably, hyponatremia can occur in both hemorrhagic and ischemic strokes, with a higher prevalence in the latter (Ehtesham et al., 2019; Fortune and Garcia-Tsao, 2013).

Among the 49 adults with hyponatremia in this cohort, 53% (*n* = 26) had mild hyponatremia, and 22.5% (*n* = 11) had severe hyponatremia. These findings are consistent with a study from Poland (Gala-Bładzińska et al., 2019) but higher than those reported in Pakistan, where 25% of patients had mild hyponatremia and 9.8% had severe hyponatremia (Mahesar et al., 2019). Conversely, our study showed a lower hyponatremia incidence compared to studies from Nigeria and two from India, where reported rates were 32.8%, 34.2%, and 45.3%, respectively (Ehtesham et al., 2019; Shah et al., 2019; Eze et al., 2022).

Alarmingly, our study cohort comprised a younger population, with a median age of 60 years (IQR, 50–74), and more than two thirds had preexisting hypertension and heart failure, major risk factors for stroke in this region (Matuja et al., 2020). In SSA, heart failure disproportionately affects younger age groups, and hypertension is a leading etiology followed by cardiomyopathy and rheumatic heart disease (Gallagher et al., 2018). Chronic hypertension leads to sustained left ventricular pressure overload, triggering structural and functional remodeling through various molecular mechanisms, ultimately resulting in diastolic dysfunction and heart failure (Yuen et al., 2022). These findings underscore the urgent need for targeted prevention strategies, including early detection, treatment, and optimal control of hypertension and other cardiovascular disorders. Further research investigating the potential causes of secondary hypertension in this younger population is warranted.

In our study, the majority of adults, 54.4% (*n* = 31/49), were treated with hypertonic solution (mannitol), which was strongly associated with hyponatremia. Yuen et al., from Arizona, USA, demonstrated similar results (Powers et al., 2018). Mannitol can cause hyponatremia and is usually prescribed in the context of treating cerebral edema; by raising plasma osmolality, it establishes a transcellular osmotic gradient, causing water to leave cells and lowering the serum sodium concentration by dilution or translocational hyponatremia (Rodrigues et al., 2014).

In the current study, NIHSS score was used to assess stroke severity and was significantly associated with the development of hyponatremia. Similar results were observed in studies done in the United States by Rodriquez et al. and Khan et al., which demonstrated that patients with hyponatremia had higher NIHSS scores on admission, during hospitalization, and at discharge (Khan et al., 2023; Rahman and Friedman, 2009). The increased disability and poor outcomes associated with hyponatremia may be attributed to the exacerbation of cerebral edema following both hemorrhagic and ischemic injuries. Pathophysiological mechanisms include the risk of developing cerebral edema, seizures, and delayed cerebral infarctions. In these patients, hyponatremia may be caused by the syndrome of inappropriate antidiuretic hormone secretion, cerebral salt wasting syndrome, or heart failure itself (Diringer et al., 1989; Liamis et al., 2020). In addition, aggressively correcting hyponatremia may lead to osmotic demyelination, further worsening patients' clinical outcomes (Kuramatsu et al., 2014).

Hyponatremia is a significant prognostic indicator in stroke patients, affecting short- and long-term outcomes in terms of disability, hospital stay, and mortality (Shah et al., 2019). In the current study, adults with hyponatremia were observed to have significantly longer hospital stays than those without hyponatremia. Similar observations were made in a previous study done in India, which demonstrated that prolonged hospital stay was associated with higher rates of complications, such as fever and infections (Shah et al., 2019; Robenolt Gray et al., 2014; Carcel et al., 2016). This finding is clinically relevant, as prolonged hospital stays are linked to poor prognosis, increased risk of hospital-acquired infections, and higher health care costs. Notably, leukocytosis, a key laboratory marker of probable infections, was significantly associated with 30-day mortality. Furthermore, prolonged hospitalization may be attributed to hyponatremia itself or its management, particularly overcorrection, which can lead to worsening neurological symptoms.

Interestingly, we did not observe a significant difference in mortality rates between patients with and without hyponatremia, a finding consistent with studies from Poland and Germany, where mortality was assessed at 90 days (Gala-Bładzińska et al., 2019; Robenolt Gray et al., 2014). However, a larger study from the United Kingdom reported a significantly higher mortality among adults with hyponatremia, although the higher proportion of hemorrhagic stroke cases in that cohort may serve as a potential confounder (Carcel et al., 2016).

These findings highlight the need for larger scale studies with extended follow-up durations to better understand the causes of hyponatremia and its impact on stroke outcomes in Tanzania. At BMC, we have recently established a dedicated stroke unit with trained multidisciplinary teams, which is expected to improve stroke care, reduce complications, and lower mortality rates.

### Study limitations

Our study is limited by the following: It was a single center with a small sample size and a cohort of patients with a high intracranial hemorrhage burden and large vessel ischemic strokes, limiting the results' generalizability. The short follow-up period made drawing definitive conclusions about hyponatremia's impact on patient outcomes difficult. In assessing hypervolemia, we were unable to measure orthostasis because our patient cohort had moderate to severe neurological impairment, with a median arrival NIHSS score of 26. Similarly, compared to previous studies (Huang et al., 2012), the relatively high median NIHSS score in our cohort, as well as the shorter follow-up duration, may have contributed to the lack of observed statistical differences in mortality between the groups. Similarly, we did not measure ocular pressure.

## Conclusion

This study highlights the significant burden of hyponatremia among adults with stroke in Northwestern Tanzania. Hyponatremia was strongly associated with increased stroke severity, prolonged hospital stays, and potential complications, although no significant difference in 30-day mortality was observed. The findings emphasize the importance of early recognition and appropriate management of hyponatremia in stroke patients to mitigate its impact on clinical outcomes. Given the high prevalence of preexisting hypertension and heart failure in this cohort, targeted preventive strategies and improved stroke care services, including the recently established stroke unit at BMC, are crucial in reducing stroke-related complications and mortality. Further large-scale studies with extended follow-up are needed to better understand the long-term impact of hyponatremia on stroke outcomes in low-resource settings.

## Data Availability

The raw data supporting the conclusions of this article will be made available by the authors, without undue reservation.

## References

[B1] Abissegue G. Yakubu S. I. Ajay A. S. Niyi-Odumosu F. A. (2024). systematic review of the epidemiology and the public health implications of stroke in Sub-Saharan Africa. J. Stroke Cerebrovasc. Dis. 33:8. 10.1016/j.jstrokecerebrovasdis.2024.107733 38663647

[B2] Atila C. Sailer C. O. Bassetti S. Tschudin-Sutter S. Bingisser R. Siegemund M.. (2021). Prevalence and outcome of dysnatremia in patients with COVID-19 compared to controls. Eur. J. Endocrinol. 184, 413–22. 10.1530/EJE-20-1374 33449918 PMC9494345

[B3] Banks J. L. Marotta C. A. (2007). Outcomes validity and reliability of the modified Rankin Scale: implications for stroke clinical trials: a literature review and synthesis. Stroke 38, 1091–1096. 10.1161/01.STR.0000258355.23810.c6 17272767

[B4] Barros A. J. Hirakata V. N. (2003). Alternatives for logistic regression in cross-sectional studies: an empirical comparison of models that directly estimate the prevalence ratio. BMC Med Res Methodol. 20:21. 10.1186/1471-2288-3-21 14567763 PMC521200

[B5] Carcel C. Sato S. Zheng D. Heeley E. Arima H. Yang J.. (2016). Prognostic significance of hyponatremia in acute intracerebral hemorrhage: pooled analysis of the intensive blood pressure reduction in acute cerebral hemorrhage trial studies. Crit. Care Med. 44, 1388–94. 10.1097/CCM.0000000000001628 26958746

[B6] Chobanian A. V. Bakris G. L. Black H. R. Cushman W. C. Green L. A. Izzo J. L.. (2003). Seventh report of the Joint National Committee on Prevention, Detection, Evaluation, and Treatment of High Blood Pressure. Hypertension 42, 1206–52. 10.1161/01.HYP.0000107251.49515.c2 14656957

[B7] Chukwudelunzu E. F. Mbonde A. (2024). Stroke care in sub-Saharan Africa: evaluating the present landscape and proposing strategies for improving outcomes. Adv. Neurol. 3:2804. 10.36922/an.2804

[B8] Diringer M. Ladenson P. W. Borel C. Hart G. K. Kirsch J. R. Hanley D. F.. (1989). Sodium and water regulation in a patient with cerebral salt wasting. Arch. Neurol. 46, 928–30. 10.1001/archneur.1989.00520440124031 2757534

[B9] Ehtesham M. Mohmand M. Raj K. Hussain T. Kavita F. Kumar B.. (2019). Clinical spectrum of hyponatremia in patients with stroke. Cureus 11:e5310. 10.7759/cureus.5310 31592365 PMC6773452

[B10] Eze C. O. Afolabi O. F. Kalu A. U. (2022). Prevalence of Hyponatremia in Acute Stroke Patients in a Federal Teaching Hospital, Abakaliki, Nigeria. West Afr J Med. 39, 1188–92. 36455196

[B11] Feigin V. L. Brainin M. Norrving B. Martins S. Sacco R. L. Hacke W.. (2022). World Stroke Organization (WSO): global stroke fact sheet 2022. Int. J. Stroke. 17, 18–29. 10.1177/17474930211065917 34986727

[B12] Feigin V. L. Stark B. A. Johnson C. O. Roth G. A. Bisignano C. Abady G. G.. (2021). Global, regional, and national burden of stroke and its risk factors, 1990-2019: a systematic analysis for the Global Burden of Disease Study 2019. Lancet Neurol. 20, 1–26. 10.1016/S1474-4422(21)00252-0 34487721 PMC8443449

[B13] Fortune B. E. Garcia-Tsao G. (2013). Hypervolemic hyponatremia: clinical significance and management. Clin. Liver Dis. 2, 109–12. 10.1002/cld.179 30992838 PMC6448632

[B14] Gabriel Ruiz-Sánchez J. Cuesta M. Gómez-Hoyos E. Cárdenas-Salas J. Rubio-Herrera M. Á. Martínez-González E.. (2022). Changes in serum creatinine levels can help distinguish hypovolemic from euvolemic hyponatremia. Medicina 25:851. 10.3390/medicina58070851 35888570 PMC9323891

[B15] Gala-Bładzińska A. Czarnota J. Kaczorowski R. Braun M. Gargasz K. Bartosik-Psujek H.. (2019). Mild hyponatremia discovered within the first 24 hours of ischemic stroke is a risk factor for early post stroke mortality. Adv. Clini. Exp. Med. 28, 1321–7. 10.17219/acem/103070 31518495

[B16] Gallagher J. McDonald K. Ledwidge M. Watson C. J. (2018). Heart Failure in Sub-Saharan Africa. Card Fail Rev. 4, 1. 10.15420/cfr.2018:4:1 29892471 PMC5971674

[B17] Hossain M. F. Kharel M. Husna A. U. Khan M. A. Aziz S. N. Taznin T.. (2023). Prevalence of electrolyte imbalance in patients with acute stroke: a systematic review. Cureus 15:e43149. 10.7759/cureus.43149 37692728 PMC10484326

[B18] Huang W. Y. Weng W. C. Peng T. I. Chien Y. Y. Wu C. L. Lee M.. (2012). Association of hyponatremia in acute stroke stage with three-year mortality in patients with first-ever ischemic stroke. Cerebrovasc. Dis. 34, 55–62. 10.1159/000338906 22759703

[B19] Karunanandham S. Rajappa T. Selvaraju K. (2018). Hyponatremia in patients admitted with stroke. J. Clini. Diagnost. Res. 12, 34–6. 10.7860/JCDR/2018/36176.11954

[B20] Khan A. Khan Z. Khan S. Ullah A. Ayub G. Tariq M. N.. (2023). Frequency of hyponatremia and its impact on prognosis in ischemic stroke. Cureus. 15:e40317. 10.7759/cureus.40317 37448406 PMC10337874

[B21] Kuramatsu J. B. Bobinger T. Volbers B. Staykov D. Lücking H. Kloska S. P.. (2014). Hyponatremia is an independent predictor of in-hospital mortality in spontaneous intracerebral hemorrhage. Stroke. 45, 1285–91. 10.1161/STROKEAHA.113.004136 24713532

[B22] Lewis J. L. (2023). MSD Manual, Hyponatremia. p. 1–12. Available online at: https://www.msdmanuals.com/professional/endocrine-and-metabolic-disorders/electrolyte-disorders/hyponatremia

[B23] Liamis G. Barkas F. Megapanou E. Christopoulou E. Makri A. Makaritsis K.. (2020). Hyponatremia in acute stroke patients: pathophysiology, clinical significance, and management options. Eur. Neurol. 82, 32–40. 10.1159/000504475 31722353

[B24] Mahesar S. A. Memon S. F. Mustafa S. Javed A. Butt S. M. (2019). Evaluation of hyponatremia in ischemic stroke patients in a tertiary care hospital of Karachi, Pakistan. Cureus. 11, e3926. 10.7759/cureus.3926 30937232 PMC6433087

[B25] Matuja S. S. Mlay G. Kalokola F. Ngoya P. Shindika J. Andrew L.. (2023). Predictors of 30-day mortality among patients with stroke admitted at a tertiary teaching hospital in Northwestern Tanzania: a prospective cohort study. Front Neurol. 13:1100477. 10.3389/fneur.2022.1100477 36742055 PMC9889987

[B26] Matuja S. S. Munseri P. Khanbhai K. (2020). The burden and outcomes of stroke in young adults at a tertiary hospital in Tanzania : a comparison with older adults. BMC Neurol. 20:206. 10.1186/s12883-020-01793-2 32450825 PMC7247244

[B27] Mckee P. A. Castelli W. P. McNamara P. M. Kannel W. B. (1971). The natural history of congestive heart failure. The Framingham study. N. Engl. J. Med. 285, 1441–6. 10.1056/NEJM197112232852601 5122894

[B28] Nishida C. Ko G. T. Kumanyika S. (2010). Body fat distribution and noncommunicable diseases in populations: Overview of the 2008. WHO Expert Consultation on Waist Circumference and Waist-Hip Ratio. Eur. J. Clini. Nutri. 64, 2–5. 10.1038/ejcn.2009.139 19935820

[B29] Okeng'o K. Chillo P. Gray W. K. Walker R. W. Matuja W. (2017). Early mortality and associated factors among patients with stroke admitted to a large teaching hospital in Tanzania. J. Stroke Cerebrovasc. Dis. 26, 871–8. 10.1016/j.jstrokecerebrovasdis.2016.10.037 27913201

[B30] Powers W. J. Rabinstein A. A. Ackerson T. Adeoye O. M. Bambakidis N. C. Becker K.. (2018). (2018). Guidelines for the early management of patients with acute ischemic stroke: a guideline for healthcare professionals from the American Heart Association/American Stroke Association. Stroke. 49, e46–110. 10.1016/j.jvs.2018.04.007 29367334

[B31] Rahman M. Friedman W. A. (2009). Hyponatremia in neurosurgical patients: clinical guidelines development. Neurosurgery. 65, 925–35. 10.1227/01.NEU.0000358954.62182.B3 19834406

[B32] Robenolt Gray J. Morbitzer K. A. Liu-DeRyke X. Parker D. Hall Zimmerman L. Rhoney D. H. (2014). Hyponatremia in patients with spontaneous intracerebral hemorrhage. J Clin Med. 3, 1322–32. 10.3390/jcm3041322 26237605 PMC4470185

[B33] Rodrigues B. Staff I. Fortunato G. McCullough L. D. (2014). Hyponatremia in the prognosis of acute ischemic stroke. J. Stroke Cerebrovass. Dis. 23, 850–4. 10.1016/j.jstrokecerebrovasdis.2013.07.011 23954607

[B34] Segal A. (2017). “Disorders of extracellular volume: hypovolemia and hypervolemia,” in. *CURRENT Diagnosis andamp; Treatment: Nephrology and Hypertension*, eds. E. V. Lerma, M. H. Rosner, M. A. Perazella (New York, NY: McGraw-Hill Education).

[B35] Shah A. Sabir S. Artani M. Salam O. Khan S. Rizwan A.. (2019). Significance of Hyponatremia as an Independent Factor in Predicting Short-term Mortality in Patients with Hemorrhagic Stroke. Cureus. 11, e4549. 10.7759/cureus.4549 31275773 PMC6592831

[B36] Shima S. Niimi Y. Moteki Y. Takahashi O. Sato S. Inoue T.. (2020). Prognostic significance of hyponatremia in acute stroke: a systematic review and meta-analysis. Cerebrovasc. Dis. 49, 531–539. 10.1159/000510751 33017822

[B37] Streng K. W. Voors A. A. Hillege H. L. Anker S. D. Cleland J. G. Dickstein K.. (2018). Waist-to-hip ratio and mortality in heart failure. Eur. J. Heart Fail. 20, 1269–77. 10.1002/ejhf.1244 29963737

[B38] Verbalis J. G. Goldsmith S. R. Greenberg A. Korzelius C. Schrier R. W. Sterns R. H.. (2013). Diagnosis, evaluation, and treatment of hyponatremia: expert panel recommendations. Am. J. Med. 126, S1–42. 10.1016/j.amjmed.2013.07.006 24074529

[B39] Walker R. Whiting D. Unwin N. Mugusi F. Swai M. Aris E.. (2010). Stroke incidence in rural and urban Tanzania: a prospective, community-based study. Lancet Neurol. 9, 786–92. 10.1016/S1474-4422(10)70144-7 20609629

[B40] Walker R. W. Jusabani A. Aris E. Gray W. K. Mugusi F. Swai M.. (2013). Correlates of short- and long-term case fatality within an incident stroke population in Tanzania. South Afric. Med. J. 103, 107–12. 10.7196/SAMJ.5793 23374304

[B41] Worthley G. Guerin M. Paint R. W. (1987). For Calculating osmolality, the simplest formula is the best. Anaesth Intens Care. 15, 199–202. 10.1177/0310057X8701500214 3605570

[B42] Yuen K. C. J. Sharf V. Smith E. Kim M. Yuen A. S. M. Macdonald P. R.. (2022). Sodium and water perturbations in patients who had an acute stroke: Clinical relevance and management strategies for the neurologist. Stroke and Vascular Neurology. BMJ Publishing Group. 7, 258–266. 10.1136/svn-2021-001230 34969834 PMC9240457

[B43] Zhang H. Wang X. Xavier O. M. J. (2010). D, Liu L, Zhang H, et al. Articles Risk factors for ischaemic and intracerebral haemorrhagic stroke in 22 countries (the INTERSTROKE study). Lancet 376, 112–23. 10.1016/S0140-6736(10)60834-3 20561675

[B44] Zou G. A. (2004). Modified poisson regression approach to prospective studies with binary data. Am. J. Epidemiol. 159, 702–706. 10.1093/aje/kwh090 15033648

