# Supplementary material for: The burden of hyponatremia and 30-day outcomes among adults admitted with stroke at a large tertiary teaching hospital in Northwestern Tanzania
Source: Front Stroke. 2025 Mar 21;4:1546358. doi: 10.3389/fstro.2025.1546358 (PMC12802786; doi:10.3389/fstro.2025.1546358)
Supplement: Supplementary file 1 [file Table_1.docx]

Supplementary file 1: Logistic regression of factors associated with hyponatremia

| Variable | Unadjusted OR (95% CI) | p-value | Adjusted OR (95% CI) | p-value |
| --- | --- | --- | --- | --- |
| Age (years) | 0.99 (0.98 - 1.02) | 0.79 | 0.99 (0.97 - 1.01) | 0.518 |
| Sex |  |  |  |  |
| Male | Ref |  |  |  |
| Female | 0.83 (0.42 - 1.66) | 0.6 |  |  |
| Comorbidities |  |  |  |  |
| Hypertension | 1.12 (0.49 - 2.52) | 0.784 |  |  |
| Diabetes | 740425970 (0 - 0) | 0.999 |  |  |
| Others | Ref |  |  |  |
| Previous medications |  |  |  |  |
| Hyponatremia associated | 0.92 (0.415 - 2.05) | 0.84 | 0.99 (0.43 - 2.28) | 0.989 |
| Others | Ref |  |  |  |
| Medication on arrival |  |  |  |  |
| Mannitol | 0.71 (0.33 - 1.55) | 0.389 |  |  |
| Others | Ref |  |  |  |
| Referral status |  |  |  |  |
| Self-referral | 0.79 (0.39 - 1.61) | 0.523 |  |  |
| Others | Ref |  |  |  |
| Health insurance |  |  |  |  |
| Insured | 1.03 (0.51 - 2.10) | 0.932 |  |  |
| Not insured | Ref |  |  |  |
| Alcohol use |  |  |  |  |
| Yes | 0.9 (0.36 - 2.20) | 0.797 |  |  |
| No | Ref |  |  |  |
| Smoking |  |  |  |  |
| Yes | 0.86 (0.28 - 2.63) | 0.797 |  |  |
| No | Ref |  |  |  |
| Salt restriction |  |  |  |  |
| Yes | 0.78 (0.39 - 1.54) | 0.473 |  |  |
| No | Ref |  |  |  |
| Pyrexia |  |  |  |  |
| Yes | 1.62 (0.18 - 14.8) | 0.672 |  |  |
| No | Ref |  |  |  |
| Type of Stroke |  |  |  |  |
| Haemorrhagic | 0.58 (0.29 - 1.14) | 0.115 | 0.59 (0.28 - 1.25) | 0.169 |
| Ischemic | Ref |  |  |  |
| NIHSS on arrival | 0.98 (0.95 - 1.01) | 0.179 | 0.99(0.95 - 1.02) | 0.344 |

NIHSS: National Institutes of Health Stroke Scale
